# Absorption wavelength along chromophore low-barrier hydrogen bonds

**DOI:** 10.1016/j.isci.2022.104247

**Published:** 2022-04-13

**Authors:** Masaki Tsujimura, Hiroyuki Tamura, Keisuke Saito, Hiroshi Ishikita

**Affiliations:** 1 Department of Advanced Interdisciplinary Studies, The University of Tokyo, 4-6-1 Komaba, Meguro-ku, Tokyo 153-8904, Japan; 2 Department of Applied Chemistry, The University of Tokyo, 7-3-1 Hongo, Bunkyo-ku, Tokyo 113-8654, Japan; 3 Research Center for Advanced Science and Technology, Graduate School of Engineering, The University of Tokyo, 4-6-1 Komaba, Meguro-ku, Tokyo 153-8904, Japan

**Keywords:** chemistry, computational chemistry, optical materials

## Abstract

In low-barrier hydrogen bonds (H-bonds), the p*K*_a_ values for the H-bond donor and acceptor moieties are nearly equal, whereas the redox potential values depend on the H^+^ position. Spectroscopic details of low-barrier H-bonds remain unclear. Here, we report the absorption wavelength along low-barrier H-bonds in protein environments, using a quantum mechanical/molecular mechanical approach. Low-barrier H-bonds form between Glu46 and *p*-coumaric acid (*p*CA) in the intermediate pR_CW_ state of photoactive yellow protein and between Asp116 and the retinal Schiff base in the intermediate M-state of the sodium-pumping rhodopsin KR2. The H^+^ displacement of only ∼0.4 Å, which does not easily occur without low-barrier H-bonds, is responsible for the ∼50 nm-shift in the absorption wavelength. This may be a basis of how photoreceptor proteins have evolved to proceed photocycles using abundant protons.

## Highlights

• The low-barrier H-bond formation is a prerequisite for proton transfer
• How the absorption wavelength changes as H^+^ moves is an open question
• The H^+^ displacement of ∼0.4 Å leads to the absorption wavelength shift of ∼50 nm
• The localization of the molecular orbitals plays a key role in the wavelength shift

## Graphical abstract

**Figure:**
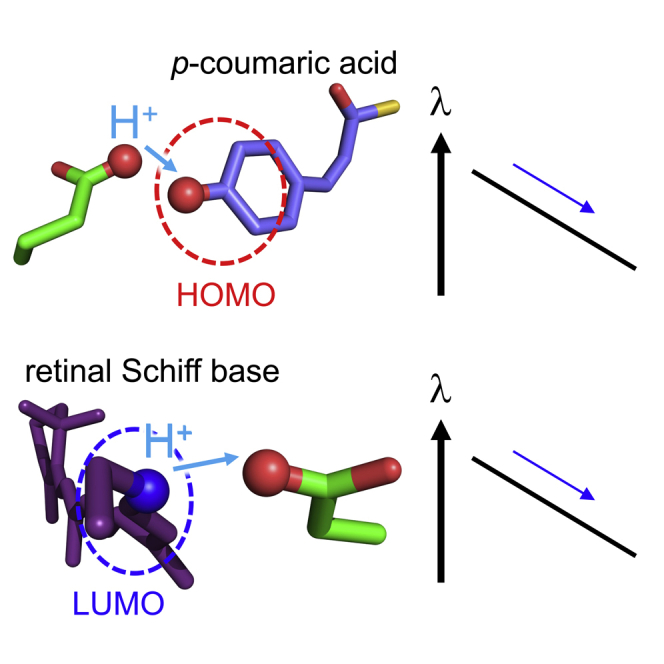

## Introduction

In biological systems, protons and electrons are abundant sources that can be used to mediate energy transfer or signal regulation. Protons can be transferred via hydrogen bonds (H-bonds) most efficiently when the p*K*_a_ values (corresponding to “proton donating power,” “acidity,” and “basicity” in the studies by Koeppe et al., 2011, 2017; Pylaeva et al., 2015) of the H-bond donor and acceptor moieties are nearly equal (p*K*_a_ difference; Δp*K*_a_ ≈ 0) (Eigen, 1964; Gerlt and Gassman, 1992). According to Frey et al. (1994), Cleland and Kreevoy (1994), and Warshel et al. (Schutz and Warshel, 2004; Warshel et al., 1995), a low-barrier H-bond can form when the “p*K*_a_ matching” occurs, facilitating proton transfer (Ishikita and Saito, 2014; Perrin and Nielson, 1997; Schutz and Warshel, 2004; Warshel et al., 1995). In the A…H^+^…B H-bond, where A and B are the H-bond partners, not the p*K*_a_ value for the release of the proton from the A…H^+^…B H-bond (i.e., p*K*_a_([A…H^+^…B]/[A…B]^−^)) but “the p*K*_a_ difference” between p*K*_a_(HA/A^−^) and p*K*_a_(HB/B^−^) is responsible for energy barrier along the H-bond (i.e., formation of a low-barrier H-bond), as indicated in the equilibrium HA + B^−^ ↔ A^−^ + B^−^ + H^+^ ↔ A^−^ + HB. For example, maleic acid (HOOC–CH=CH–COOH) has two p*K*_a_ values (1.9 and 6.6). A low-barrier H-bond –COO^−^…H^+^…^−^OOC– forms upon the first deprotonation of –COOH HOOC– (p*K*_a_ = 6.6). Deprotonation of –COO^−^…H^+^…^−^OOC– to –COO^−^…^−^OOC– (i.e., p*K*_a_([A…H^+^…B]/[A…B]^−^)) occurs with p*K*_a_ = 1.9 for the second deprotonation, which is not relevant to the p*K*_a_ value of ∼4 (e.g., 4.4 for acrylic acid) for the two H-bond donor/acceptor moieties (i.e., p*K*_a_(HA/A^−^) and p*K*_a_(HB/B^−^)).

The shape of the potential energy curve of a low-barrier H-bond is symmetric because p*K*_a_(donor) ≈ p*K*_a_(acceptor), whereas that of a standard H-bond is asymmetric. Indeed, theoretical studies by Ikeda et al. showed that the difference in the original p*K*_a_ values between the donor and acceptor groups is directly correlated with the energy difference between the two H-bond moieties (Ikeda et al., 2017). The simplest way to match the p*K*_a_ values and form a low-barrier H-bond is to use identical groups as the H-bond donor and acceptor. This condition allows the easy occurrence of proton transfer via low-barrier H-bond species H_2_O…H^+^…OH_2_ in the bulk water region. In contrast, the formations of low-barrier H-bonds between chemically different groups are often observed in protein environments. In such cases, electrostatic interactions with the protein environment equate the p*K*_a_ values (proton donating abilities) of the two moieties. In contrast, salt bridges can form when the p*K*_a_ difference is large. Salt bridges are strong in polar environments and play a key role in electrostatic interactions at the protein interface (Ishikita and Saito, 2014).

**Figure 1:**
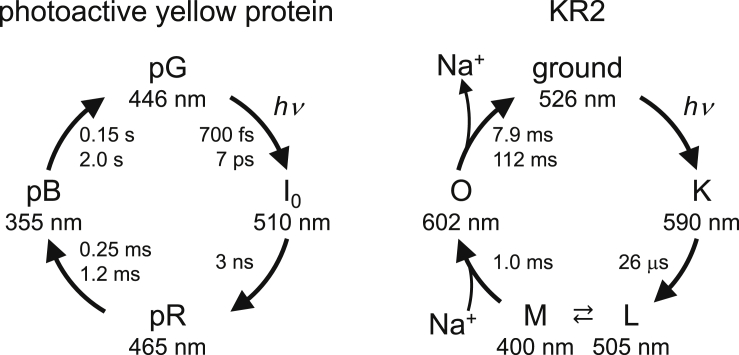
Photocycle intermediate states in photoactive yellow protein and KR2

In the light-driven sodium-pumping rhodopsin KR2, the intermediate L- and M-states (505 and 400 nm, respectively), where the chromophore retinal Schiff base is protonated and deprotonated, reach equilibrium 26 μs after the light irradiation (Inoue et al., 2013) (Figure 1). The crystal structures of the two states are not reported, whereas time-resolved X-ray free electron laser (XFEL) structures of KR2 were reported recently (Skopintsev et al., 2020). In the low-pH structure (crystalized at pH 4 and soaked at pH 8) of KR2 (Kato et al., 2015), no groups donate H-bonds to the counterion Asp116, increasing p*K*_a_(Asp116). In this structure, the retinal Schiff base and Asp116 form a low-barrier H-bond (Tsujimura and Ishikita, 2021). The absorption wavelength calculated using the low-pH structure is low (416 nm) and reproduces the M-state absorption wavelength (400 nm [Inoue et al., 2013]), which suggests that the low-pH structure is likely to represent the M-state geometry (Tsujimura and Ishikita, 2021). Thus, the L- to M-state transition may proceed via proton transfer along the low-barrier H-bond. The H^+^-released M-state induces Na^+^ binding, leading to res the formation of the O-state.

It was previously proposed that in the photoactive yellow protein, a low-barrier H-bond existed between the chromophore *p*-coumaric acid (*p*CA) and Glu46 in the ground state, pG (Yamaguchi et al., 2009). However, many theoretical and experimental studies have suggested that *p*CA and Glu46 do not form a low-barrier H-bond in the ground state (Graen et al., 2016; Saito and Ishikita, 2012a, 2012b, 2013; Thomson et al., 2019; Wang, 2019; Yoshimura et al., 2017) (Figure 1). Nearly, all of new experimental and theoretical studies have consistently shown that the H-bond is not a low-barrier H-bond, as recently reviewed in the study by Wang, 2019. (Highly unstable) deprotonated Arg52 on the protein bulk surface, which was initially proposed as “experimental evidence” of the presence of a low-barrier H-bond in the neutron diffraction study (Yamaguchi et al., 2009), has also been denied in the careful reinvestigation of the original diffraction data (Graen et al., 2016). Thus, a low-barrier H-bond cannot be defined by only the bond length, the H atom position, or strength of an H-bond: identification of a low-barrier H-bond can be valid only if the shape of the potential energy profile of the H-bond is symmetric (i.e., the p*K*_a_ values for the two moieties are nearly equal), as suggested by Schutz and Warshel (2004).

In the ground state, Tyr42 donates an H-bond to *p*CA, which decreases p*K*_a_(*p*CA) with respect to p*K*_a_(Glu46), i.e., p*K*_a_(*p*CA) < p*K*_a_(Glu46) (Saito and Ishikita, 2012a). Spectroscopic studies using model systems suggested that the donation of an H-bond to *p*CA from Tyr42 and the low polarity of the protein environment with respect to aqueous solution contribute to p*K*_a_(*p*CA) < p*K*_a_(Glu46) (Koeppe et al., 2013, 2021). However, electronic excitation results in an intermediate (pR_CW_) structure (Ihee et al., 2005), forming a low-barrier H-bond between *p*CA and Glu46 owing to the loss of the H-bond donation from Tyr42 to *p*CA (Saito and Ishikita, 2013). The formation of a low-barrier H-bond in the pR_CW_ structure is consistent with the observation of proton transfer from protonated Glu46 to *p*CA in the pR state (Xie et al., 1996). The pR state is unstable as proton transfer proceeds, leading to large structural changes in the *p*CA chromophore (Schotte et al., 2012) and formation of the pB state. The absorption wavelength is shortened by 110 nm during the pR (465 nm (Hoff et al., 1994)) to pB (355 nm (Hoff et al., 1994)) transition. The signaling state of the protein is the pB state, which has the shortest absorption wavelength among the photocycle intermediate states (Cusanovich and Meyer, 2003; Hellingwerf et al., 2003).

Spectroscopic studies showed that changes in the absorption wavelength were observed in response to the H^+^ migration toward the H-bond partner using the model systems (Koeppe et al., 2011). Hereby, the influence of the H^+^ position on UV/vis spectra was investigated by replacing the acceptor group. In the H-bonds between phenol derivatives (Ph) and anions (X^−^), dual UV-vis absorption peaks derived from [Ph–H…X^−^] and [Ph^−^…H–X] were observed (Koeppe et al., 2011), which indicates that H^+^ localizes at the proton donor and acceptor moieties (Koeppe et al., 2011, 2017; Pylaeva et al., 2015). In practice, the proton vibrational functions are delocalized, solvent fluctuations are present, and only average O-H distances and UV-vis spectra can be measured experimentally (e.g., Koeppe et al., 2011, 2017; Pylaeva et al., 2015). Thus, it might be a great challenge to simulate the experimental spectra computationally. On the other hand, the maximum absorption wavelengths in the chromophores of the proteins can be calculated appropriately, as demonstrated for microbial rhodopsins (Tsujimura and Ishikita, 2020, 2021; Tsujimura et al., 2021a, 2021b).

Here we look for chromophore H-bonds and investigate how the electronic structure and the absorption wavelength of the chromophore change owing to the H^+^ migration using a quantum mechanical/molecular mechanical (QM/MM) approach.

## Results and discussion

**Figure 2:**
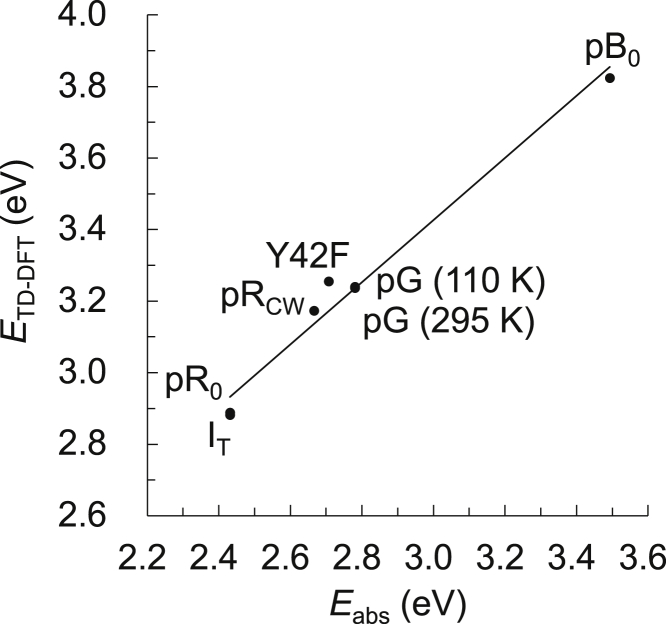
Correlation between the excitation energy calculated using the TD-DFT method (*E*_TD-DFT_) and the experimentally measured absorption energy (*E*_abs_); coefficient of determination *R*^2^ = 0.977 (0.956 when excluding the pB_0_ structure) The experimentally measured absorption energies correspond to 446 nm (Meyer, 1985) for pG, 510 nm (Ujj et al., 1998) for I_T_ and pR_0_ (both corresponding to the I_0_ state), 465 nm (Hoff et al., 1994) for pR_CW_, 355 nm (Hoff et al., 1994) for pB_0_, and 458 nm for Y42F (Brudler et al., 2000). The excitation energy for pR_CW_ was calculated in the presence of protonated Glu46 (O_Glu46_-H…O_*p*CA_).

Using the time-dependent density functional theory (TD-DFT) method, we calculated the excitation energies for the photocycle intermediate states (pG [295 K], pG [110 K], I_T_, pR_0_, pR_CW_, and pB_0_) and the Y42F mutant of photoactive yellow protein. The calculated excitation energy, *E*_TD-DFT_ (eV), has a high correlation with the experimentally determined energy, *E*_abs_ (eV) (Figure 2, *R*^2^ = 0.977), and is best described by the following equation:

(Equation 1) $$E_{\text{abs}}=1.149\;E_{\mathrm{TD}-\mathrm{DFT}}-0.939.$$

**Figure 3:**
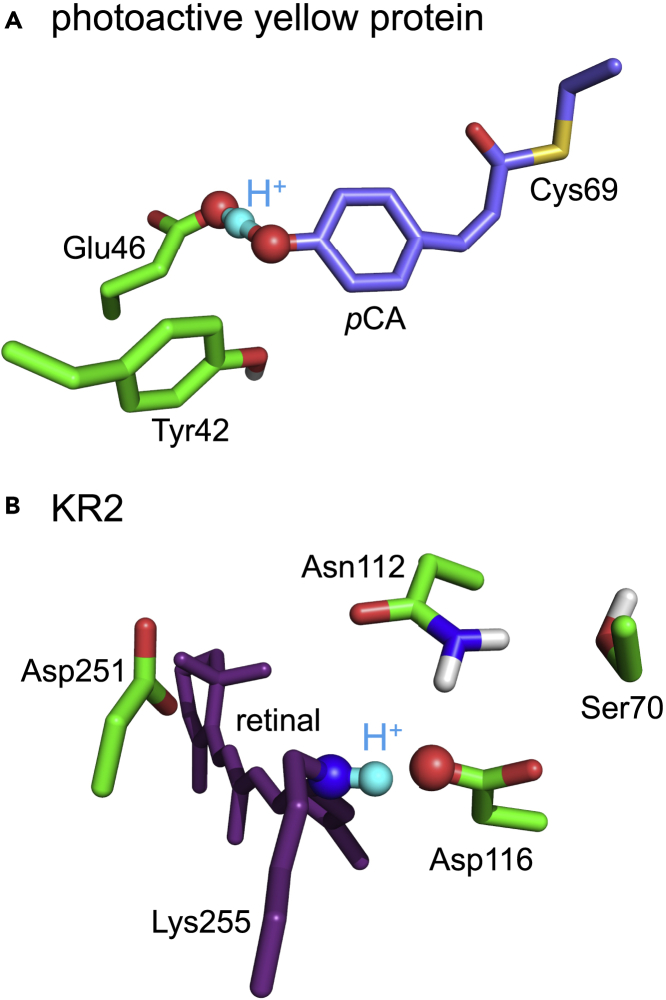
Overview of low-barrier H-bonds in the photoactive yellow protein and KR2 structures (A) H-bond network of *p*CA in the QM/MM-optimized intermediate pR_CW_ structure (PDB ID: 1TS7 (Ihee et al., 2005)). (B) H-bond network of the retinal Schiff base in the QM/MM-optimized KR2 structure (PDB ID: 6TK3 (Skopintsev et al., 2020)). Cyan balls indicate H^+^ in the low-barrier H-bonds.

**Figure 4:**
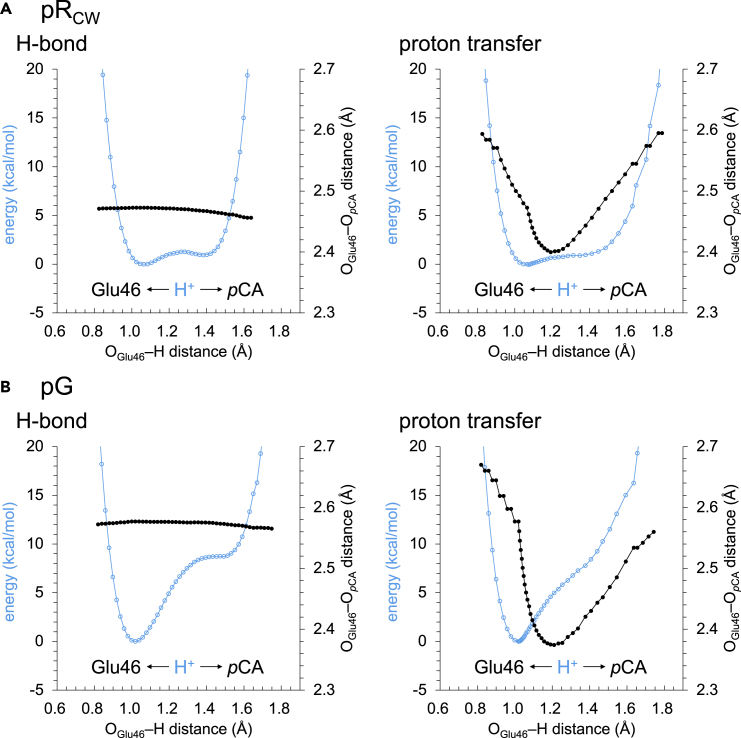
Potential energy profile for H-bonds and proton transfer in photoactive yellow protein (A) pR_CW_ (Ihee et al., 2005) state. (B) pG (Anderson et al., 2004) state. . Cyan open circles indicate energies. Black closed circles indicate O_Glu46_…O_*p*CA_ distance. In the potential energy profile for H-bonds, the H-bond donor…acceptor distance remains unchanged in response to the H^+^ movement. In the potential energy profile for proton transfer, the H-bond donor...acceptor distance changes in response to the H^+^ movement.

The shape of the potential energy profile for the H-bond (i.e., the H-bond donor and acceptor distance remains unchanged in response to the H^+^ movement, Figures 3, 4A, and S2A and Table S1) is symmetric, which indicates that a low-barrier H-bond forms between Glu46 and *p*CA in the intermediate pR_CW_ structure, as suggested in the QM/MM calculations (Saito and Ishikita, 2013). The low-barrier H-bond formation suggests that the release of the H^+^ from Glu46 to *p*CA occurs easily. The shape of the potential energy profile for proton transfer (i.e., the H-bond donor and acceptor distance changes in response to the H^+^ movement) is also symmetric, which conforms that a low-barrier H-bond forms in the pR_CW_ structure (Figures 4A and S2B and Table S1). In contrast, the shape of the potential energy profile for the corresponding H-bond is asymmetric in the ground pG state (Figure 4B), which suggests that the H-bond is not a low-barrier H-bond in the pG state (Saito and Ishikita, 2012a, 2013).

**Figure 5:**
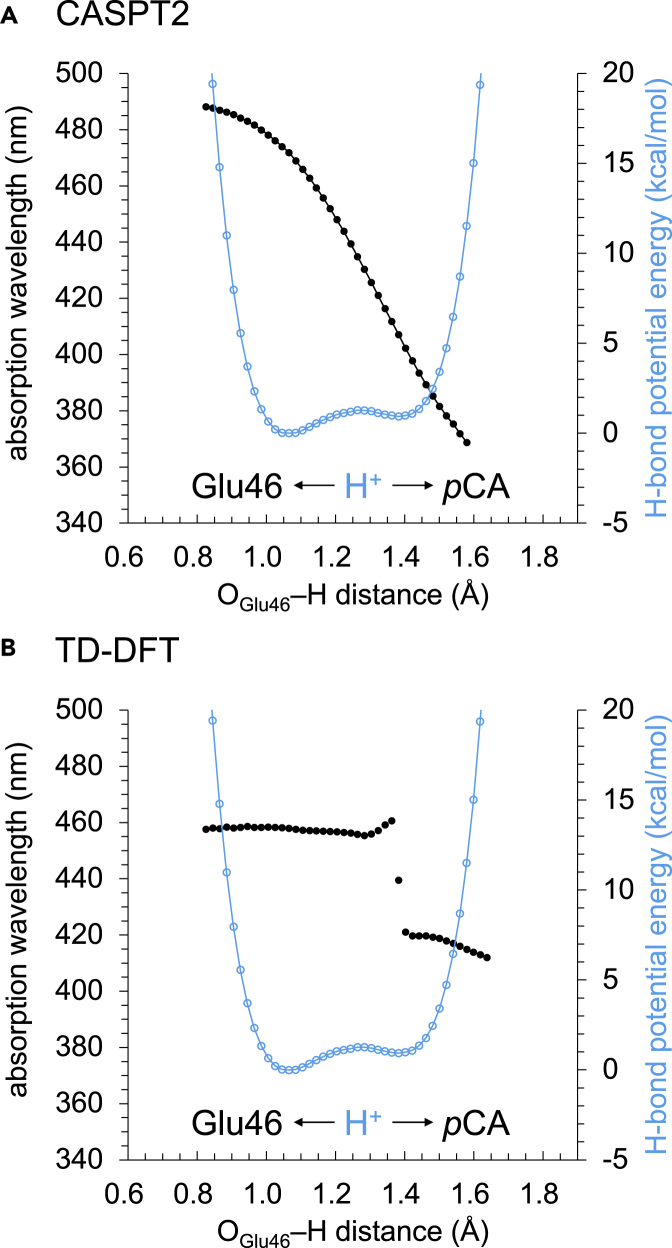
Absorption wavelength of *p*CA along the low-barrier H-bond in the pR_CW_ structure of photoactive yellow protein (A) CASPT2 calculation. (B) TD-DFT calculation. Black circles indicate absorption wavelengths, and cyan open circles indicate H-bond energies.

The excitation from the highest occupied molecular orbital (HOMO) to the lowest unoccupied molecular orbital (LUMO) is the main characteristic of the excited state of *p*CA. When H^+^ is located at the Glu46 moiety (i.e., before the proton transfer), the calculated absorption wavelength is ∼470 nm (Figure 5A, using the the second-order perturbation theory (CASPT2) method) or ∼460 nm (Figure 5B, using the TD-DFT method), which is consistent with the experimentally determined absorption wavelength of 465 nm for the pR state (Hoff et al., 1994).

Intriguingly, as the H^+^ approaches the *p*CA moiety along the low-barrier H-bond, the absorption wavelength decreases from 460–470 nm to 410–420 nm (Figure 5). The absorption wavelength decreases continuously when calculated using the CASPT2 method (Figure 5A) and discontinuously when calculated using the TD-DFT method (Figure 5B). The observed decrease of ∼50 nm in the absorption wavelength is comparable to the decrease of 51 nm observed upon the protonation of isolated *p*CA in water (Putschögl et al., 2008).

**Figure 6:**
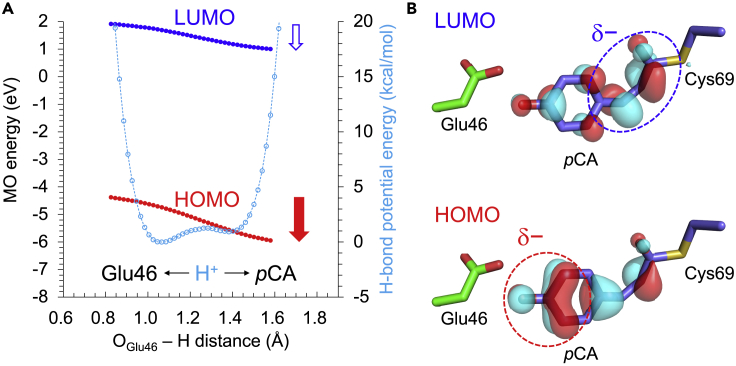
Molecular orbitals (MOs) in the low-barrier H-bond in the pR_CW_ structure of photoactive yellow protein (A) Energy levels of LUMO and HOMO of *p*CA calculated using the CASSCF method. Blue and red closed circles indicate the energy levels of LUMO and HOMO, respectively. The blue and red arrows indicate the stabilizations of LUMO and HOMO energy levels owing to the migration of H^+^, respectively. Cyan open circles indicate H-bond energies. (B) Locations of LUMO and HOMO in *p*CA.

The LUMO energy level of *p*CA is not significantly affected by the H^+^ position (Figure 6A). On the other hand, the HOMO energy level decreases more significantly than the LUMO energy level as the H^+^ approaches the *p*CA moiety. This behavior is due to the localization of LUMO at the Cys69 moiety and the localization of HOMO at the protonation site (hydroxyl group moiety) (Figure 6B). Thus, the absorption wavelength (HOMO to LUMO) decreases as the H^+^ approaches the *p*CA moiety along the low-barrier H-bond (Figure 6).

Notably, there are several limitations of TD-DFT (e.g., Barbatti and Crespo-Otero, 2014). The discontinuous decrease in the absorption wavelength observed in the TD-DFT calculation (Figure 5B) is likely an artifact of the methodology. The excited state can include double excitations that are not properly described by single-excitation TD-DFT. The question about adequate description of charge-transfer is still open even with the CAM-B3LYP functional (Yanai et al., 2004).

The presence of the discontinuous decrease in the absorption wavelength observed in the TD-DFT calculation is not due to structural changes induced by the H^+^ displacement since the planarity of the double bond and the thioester region in *p*CA remains unchanged (Figure S3). The discontinuous decrease in the absorption wavelength is also observed even when the functional/basis set is replaced (e.g., the CAM-B3LYP functional, Yanai et al., 2004, Figure S4), or the H-bond donor and acceptor distance can change in response to the H^+^ movement (the potential energy profile for proton transfer, Figure S5), as far as TD-DFT is employed.

**Figure 7:**
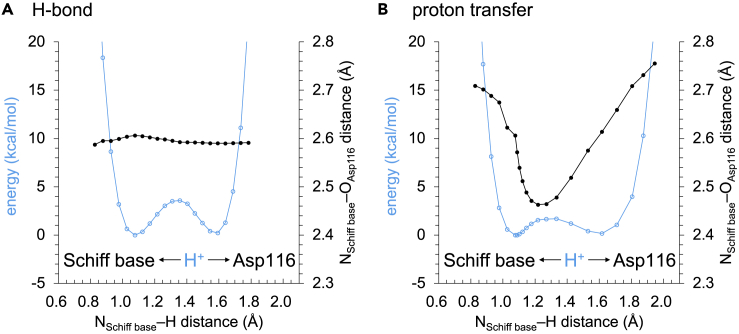
Potential energy profile for H-bonds and proton transfer in the intermediate state of KR2 (A) H-bonds.. (B) proton transfer. Cyan open circles indicate energies. Black closed circles indicate N_Schiff base_…O_Asp116_ distance. In the potential energy profile for H-bonds, the H-bond donor…acceptor distance remains unchanged in response to the H^+^ movement. In the potential energy profile for proton transfer, the H-bond donor...acceptor distance changes in response to the H^+^ movement.

In the time-resolved XFEL structure of KR2 (Skopintsev et al., 2020) obtained 30 μs or 150 μs after the light irradiation, a low-barrier H-bond forms between the retinal Schiff base and Asp116 when Ser70 does not donate an H-bond to Asp116 (Figures 7, S6, and S7 and Table S2), as suggested in recent theoretical studies (Tsujimura and Ishikita, 2021). This is consistent with the fact that the intermediate L-state (with protonated Schiff base) and M-state (with deprotonated Schiff base) reach equilibrium 26 μs after the light irradiation (Inoue et al., 2013) (Figure 1).

**Figure 8:**
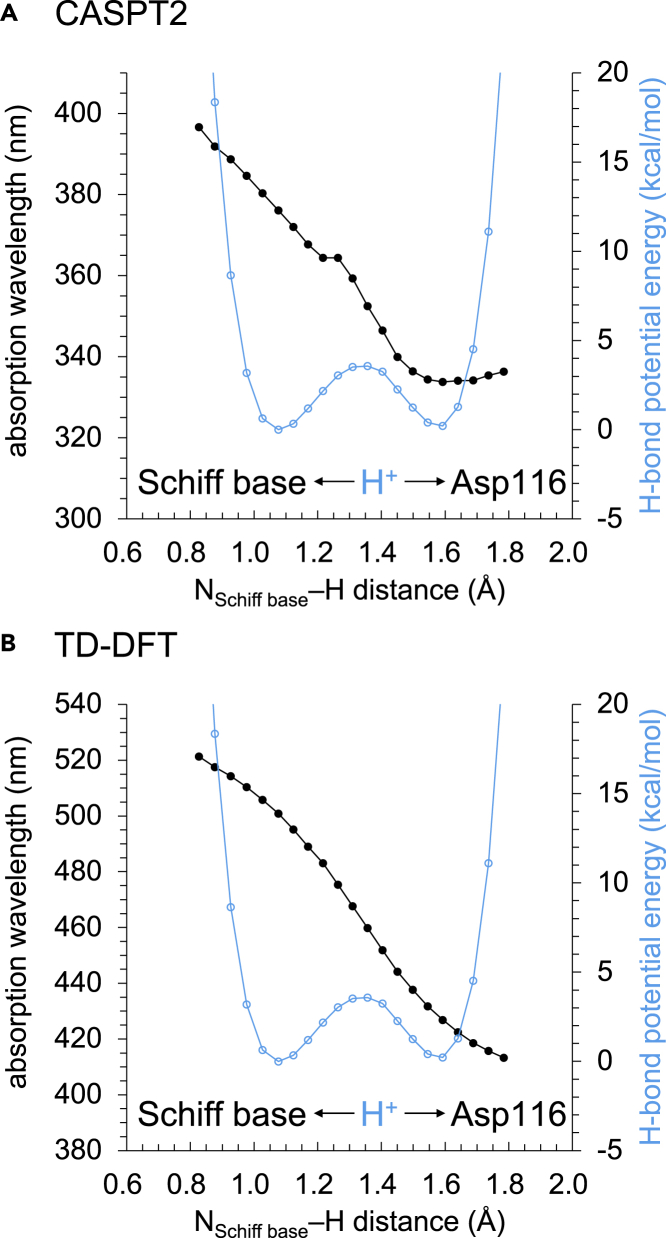
Absorption wavelength of the retinal Schiff base along the low-barrier H-bond in the KR2 structure (A) CASPT2 calculation. (B) TD-DFT calculation. Black circles indicate absorption wavelengths, and cyan open circles indicate H-bond energies.

In both CASPT2 and TD-DFT calculations of KR2, the absorption wavelength decreases continuously as the H^+^ approaches the Asp116 moiety along the low-barrier H-bond (Figure 8). In the low-pH structure (crystalized at pH 4 and soaked at pH 8) of KR2 (Kato et al., 2015), the retinal Schiff base and Asp116 form a low-barrier H-bond (Tsujimura and Ishikita, 2021). The continuous decrease in the absorption wavelength is also observed along the low-barrier H-bond in the low-pH structure when calculated using the TD-DFT method (Figure S8). The similarity in the H-bond energy and absorption wavelength profiles between the time-resolved XFEL structure (Figure 8) and the low-pH structure (Figure S8) confirms that the low-pH structure has the characteristic of the M-state, as suggested in recent theoretical studies (Tsujimura and Ishikita, 2021).

**Figure 9:**
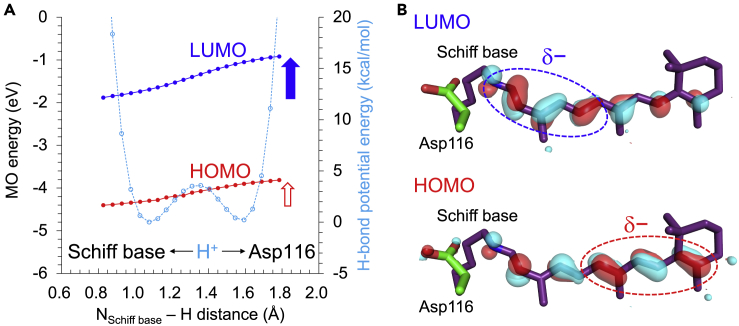
Molecular orbitals (MOs) in the low-barrier H-bond in the KR2 structure (A) Energy levels of LUMO and HOMO of the retinal Schiff base calculated using the TD-DFT method. Blue and red circles indicate the energy levels of LUMO and HOMO, respectively. The blue and red arrows indicate the destabilizations of LUMO and HOMO energy levels owing to the migration of H^+^, respectively. Cyan open circles indicate H-bond energies. (B) Locations of LUMO and HOMO in the retinal Schiff base.

The excitation from the HOMO to the LUMO is the main characteristic of the excited state of retinal Schiff base. The HOMO energy level of the retinal Schiff base remains essentially constant (Figure 9A). On the other hand, the LUMO energy level increases more significantly than the HOMO energy level as the H^+^ approaches the Asp116 moiety (Figure 9A). This behavior is attributed to the localization of LUMO at the protonation site (Schiff base moiety) and the localization of HOMO at the *β*-ionone ring moiety (Figure 9B). As a result, the absorption wavelength (HOMO to LUMO) continuously decreases as the H^+^ approaches the Asp116 moiety along the low-barrier H-bond (Figure 8).

The absorption wavelength of ∼500 nm for the protonated Schiff base calculated using the TD-DFT method is close to the experimentally measured value of 505 nm for the L-state (Inoue et al., 2013). The calculated absorption wavelength of ∼430 nm for the deprotonated Schiff base is also comparable to the experimentally measured value of 400 nm for the M-state (Inoue et al., 2013) (Figure 8B).

In contrast, the absorption wavelengths calculated using the CASPT2 method (Figure 8A, ∼380 nm for the protonated Schiff base and ∼330 nm for the deprotonated Schiff base) are significantly short with respect to the measured values (Inoue et al., 2013). The same tendency was also observed in previous studies by Pedraza-González et al., as the absorption wavelength of KR2 calculated using the CASPT2 method was ∼120 nm shorter than the measured value (Nakajima et al., 2021; Pedraza-González et al., 2019). Only when they intentionally protonated Asp251, they were able to obtain the experimentally measured absorption wavelength. However, protonation of Asp251 is energetically unlikely because it exists near the positively charged Schiff base (3.8 Å, Skopintsev et al., 2020) and Arg109 (2.7 Å, Skopintsev et al., 2020). In the present study, the calculated p*K*_a_ value for Asp251 is −1.3. In addition, no bands are observed in the protonated carboxylate region (1700–1750 cm^−1^) in Fourier-transform infrared spectroscopy (Ono et al., 2014). Furthermore, the time-resolved XFEL structures of KR2 show that Asp251 binds a positive charged sodium ion in the intermediate state (Skopintsev et al., 2020). These results suggest that Asp251 is deprotonated. Consistently, the absorption wavelength of KR2 calculated using the TD-DFT method is comparable with the measured value when Asp251 is deprotonated (Figure 8B) (Tsujimura and Ishikita, 2020).

### Conclusions

**Figure 10:**
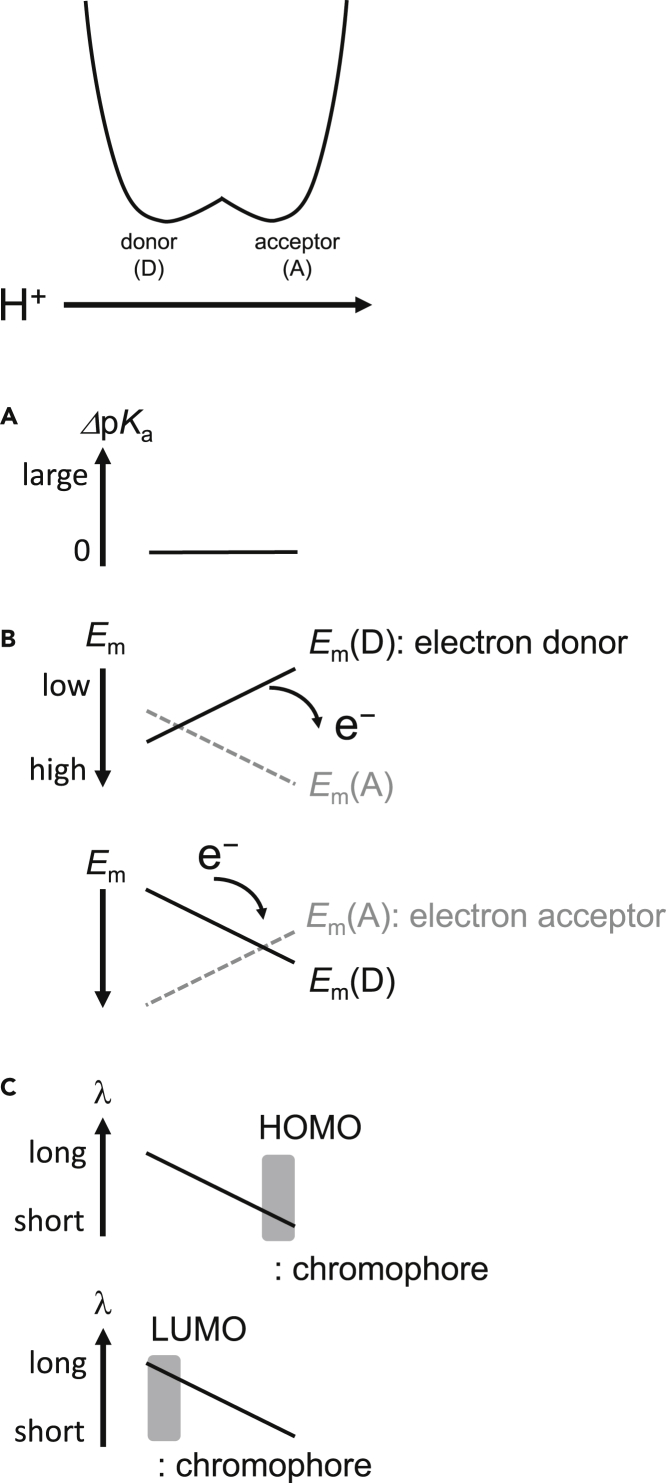
Properties of low-barrier H-bonds between chemically different groups in protein environments (A) p*K*_a_ difference (*Δ*p*K*_a_) between the H-bond donor (D) and acceptor (A) moieties (Perrin and Nielson, 1997; Warshel et al., 1995). (B) *E*_m_ for the H-bond donor (*E*_m_(D)) and acceptor (*E*_m_(A)) moities (Saito et al., 2020). (Top) H-bond donor as an electron donor (e.g., TyrZ in photosystem II (Kawashima et al., 2018; Saito et al., 2011, 2020)). The solid line indicates *E*_m_ for the H-bond donor, which serves as an electron donor. The dotted gray line indicates *E*_m_ for the H-bond acceptor. The curved arrow indicates electron transfer from the electron donor (H-bond donor). (Bottom) H-bond acceptor as an electron acceptor (e.g., Q_B_ in photosystem II (Saito et al., 2013, 2020)). The solid line indicates *E*_m_ for the H-bond acceptor, which serves as an electron acceptor. The dotted gray line indicates *E*_m_ for the H-bond donor. The curved arrow indicates electron transfer to the electron acceptor (H-bond acceptor). (C) Absorption wavelength of the chromophore. (Top) The HOMO is localized at the protonation site of the chromophore (e.g., *p*CA). (Bottom) The LUMO is localized at the protonation site of the chromophore (e.g., retinal Schiff base).

The following two characteristics have already been reported for low-barrier H-bonds: **(1) p*K***_**a**_**.** p*K*_a_(donor) and p*K*_a_(acceptor) are nearly equal along a low-barrier H-bond, facilitating proton transfer to the acceptor moiety (Perrin and Nielson, 1997; Warshel et al., 1995) (Figure 10A). **(2) Redox potential (*E***_**m**_**).** As the H^+^ transfers along a low-barrier H-bond, *E*_m_(donor) decreases and *E*_m_(acceptor) increases continuously, facilitating electron transfer to the acceptor moiety (Saito et al., 2020) (Figure 10B). Based on the findings presented here, we are able to report another characteristic of low-barrier H-bonds, i.e., **(3) absorption wavelength** (Figure 10C). If the HOMO is localized at the protonation site of the chromophore (e.g., *p*CA), the absorption wavelength shortens as H^+^ reaches the unprotonated chromophore moiety (Figure 10C, top panel). If the HOMO is located away, but the LUMO is located at the protonation site of the chromophore (e.g., retinal Schiff base), the absorption wavelength lengthens as H^+^ reaches the unprotonated chromophore moiety (Figure 10C, bottom panel). Changes in the absorption wavelength along the H-bond should also occur in standard H-bonds in response to the H^+^ movement, although H^+^ is predominantly localized at the H-bond donor moiety.

The mechanism of the absorption wavelength shifts during the photocycle is often oversimplified and is explained as “structural change” or “conformational change” especially when the molecular mechanism is unclear. Hence, the definition of “structural (conformational) change” is ambiguous. The present result shows that the H^+^ displacement of only ∼0.4 Å, which does not easily occur without low-barrier H-bonds, is responsible for the ∼50-nm shift in the absorption wavelength (Figures 5 and 8). That is, “the H^+^ displacement of only ∼0.4 Å” mainly corresponds to “structural (conformational) change.”

These findings may provide insights to help understand how photoactive proteins have evolved to control chromophore energetics using abundant protons and design the photocycle by tuning the chromophore electronic states.

### Limitations of the study

The results depend on the original atomic coordinates of the crystal structures. The original side-chain orientations may affect the results, although the geometries of the chromophore moieties are quantum-chemically optimized.

## STAR★Methods

### Key resources table

**Table:** 

REAGENT or RESOURCE	SOURCE	IDENTIFIER
**Software and algorithms**		

CHARMM	Brooks et al. (1983)	RRID:SCR_014892; https://www.charmm.org
MEAD	Bashford and Karplus (1990)	https://doi.org/10.1016/0022-2836(92)91009-E
Karlsberg	Rabenstein and Knapp (2001)	http://agknapp.chemie.fu-berlin.de/karlsberg_old/
Qsite	Murphy et al. (2000); Philipp and Friesner (1999)	https://www.schrodinger.com/products/qsite
GAMESS	Schmidt et al. (1993)	RRID:SCR_014896; http://www.msg.chem.iastate.edu/gamess/

### Resource availability

#### Lead contact

Further information and requests for resources should be directed to and will be fulfilled by the lead contact, Hiroshi Ishikita (hiro@appchem.t.u-tokyo.ac.jp).

#### Materials availability

This study did not generate new unique reagents.

### Method details

#### Coordinates and atomic partial charges

The atomic coordinates of photoactive yellow protein were taken from the X-ray structures of the wild type (PDB ID: 1OTB (Anderson et al., 2004) at 295 K and 1OT9 (Anderson et al., 2004) at 110 K) and Y42F mutant (1F9I (Brudler et al., 2000)) ground states and the Laue diffraction structures of intermediates, i.e., I_T_ (4I38 (Jung et al., 2013)), pR_0_ (4B9O (Schotte et al., 2012)), pR_CW_ (1TS7 (Ihee et al., 2005)), and pB_0_ (4BBV (Schotte et al., 2012)). The atomic coordinates of KR2 were taken from the time-resolved XFEL structure (PDB ID: 6TK3 (Skopintsev et al., 2020)). In photoactive yellow protein, crystal water molecules near *p*CA in the pB_0_ structure were included explicitly (Figure S1). Other water molecules were considered implicitly (see below) because no water molecules are identified in the I_T_ (Jung et al., 2013) and pR_CW_ (Ihee et al., 2005) structures. In KR2, all crystal water molecules were included explicitly in the calculations if not otherwise specified. During the optimization of hydrogen atom positions with the CHARMM all-atom force field (Brooks et al., 1983), the positions of all heavy atoms were fixed. In the pB_0_ structure of photoactive yellow protein, Glu46 was ionized and *p*CA was protonated (Xie et al., 1996), whereas in other structures, Glu46 was protonated and *p*CA was ionized (Ishikita and Saito, 2014; Saito and Ishikita, 2012a, 2012b). Other titratable groups were kept in their standard protonation states (i.e., acidic groups were deprotonated, and basic groups were protonated). The atomic partial charges of the amino acids were obtained from the CHARMM22 (MacKerell et al., 1998) parameter set.

#### Protonation pattern

The protonation pattern in KR2 was determined based on the electrostatic continuum model, solving the linear Poisson–Boltzmann equation with the MEAD program (Bashford and Karplus, 1990). The difference in electrostatic energy between the two protonation states, i.e., protonated and deprotonated states, in a reference model system was calculated using a known experimentally measured p*K*_a_ value (e.g., 4.0 for Asp (Nozaki and Tanford, 1967)). Accordingly, such difference in the p*K*_a_ value of the protein relative to the reference system was added to the known reference p*K*_a_ value. The experimentally measured p*K*_a_ values employed as references were 12.0 for Arg, 4.0 for Asp, 9.5 for Cys, 4.4 for Glu, 10.4 for Lys, 9.6 for Tyr (Nozaki and Tanford, 1967), and 7.0 and 6.6 for the N_ε_ and N_δ_ atoms of His, respectively (Tanokura, 1983a, 1983b, 1983c). All other titratable sites were fully equilibrated to the protonation state of the target site during titration. The dielectric constants were set to 4 inside the protein and 80 for water to use Equation 4 for KR2 (Tsujimura and Ishikita, 2020, 2021) (see below). Note that the dielectric constant of 4 for protein interior was consistently used in previous studies for microbial rhodopsins (Tsujimura and Ishikita, 2020, 2021; Tsujimura et al., 2021b). All water molecules were considered implicitly. All computations were performed at 300 K and pH 7.0 with an ionic strength of 100 mM. The linear Poisson–Boltzmann equation was solved using a three-step grid-focusing procedure at resolutions of 2.5, 1.0, and 0.3 Å. The ensemble of the protonation patterns was sampled using the Monte Carlo (MC) method with the Karlsberg program (Rabenstein and Knapp, 2001). The MC sampling yielded the probabilities of the two protonation states (protonated and deprotonated states) of the molecule.

#### QM/MM calculations

The geometry was optimized using a QM/MM approach. The restricted DFT method was employed with the B3LYP functional and LACVP∗∗+ basis sets using the QSite (Murphy et al., 2000; Philipp and Friesner, 1999; QSite, 2012) program. In photoactive yellow protein, the QM region was defined as the entireties of *p*CA, Glu46, Thr50, and Cys69 and the side chain of Tyr42. Additionally, a water molecule near Tyr42 and Glu46 was included in the QM region for the calculation of pB_0_ (Figure S1). In KR2, the QM region was defined as the retinal Schiff base (including the side chain of Lys255); side chains of Ser70, Arg109, Asn112, Trp113, Asp116, Tyr218, Asp251, and Ser254; and the water molecule near the Schiff base (H_2_O-406). All atomic coordinates were fully relaxed in the QM region. In the MM region, the positions of the H atoms were optimized using the OPLS2005 force field (Jorgensen et al., 1996), whereas the positions of the heavy atoms were fixed.

To obtain the potential energy profiles of the H-bonds, the QM/MM-optimized geometry was used as the initial geometry. The H atom under investigation was moved from the H-bond donor atom (D) toward the acceptor atom (A) by 0.02 Å for photoactive yellow protein and 0.05 Å for KR2, after which the geometry was optimized by constraining the D–H and H–A distances (for H-bond) or the H–A distance (for proton transfer), and the energy was calculated. These procedures were repeated until the H atom reached the A atom. All atomic coordinates were fully relaxed in the QM region, whereas only the H atom positions were optimized in the MM region. After the geometry optimization, the QM region was then redefined to include the H^+^ acceptor chromophore and H^+^ donor residue (i.e., *p*CA, the side chain of Cys69, and the side chain of Glu46 in photoactive yellow protein, retinal Schiff base and the side chain of Asp116 in KR2), and the absorption energies were calculated.

*i) TD-DFT.* The excitation energies, transition oscillator strengths, and energy levels of the molecular orbitals (MOs) were calculated for 20 excited states using the TD-DFT method. The B3LYP functional and 6-31G∗∗ (photoactive yellow protein) or 6-31G∗ (KR2) basis sets was employed using the GAMESS program (Schmidt et al., 1993). To determine the maximum excitation energy, we constituted an absorption spectrum *f*(*E*) as a summation of the individual absorption spectra *g*_*n*_(*E*) of each excitation state with inhomogeneous broadening described by the Gaussian function:

(Equation 2) $$f\left(E\right)=\sum_{n=1}^{20}g_{n}\left(E\right)$$

(Equation 3) $$g_{n}\left(E\right)=f_{1n}\;\exp\left[-\frac{{\left(E-E_{n}\right)}^{2}}{2c^{2}}\right]$$

where *f*_1*n*_ is the oscillator strength, *E*_*n*_ is the excitation energy of the *n*-th excited state, and *c* is a standard deviation (0.2 eV). When *f*(*E*) takes the maximum value, *E* is considered as the maximum value for the excitation energy, i.e., *E*_TD-DFT_. In KR2, the absorption energy (*E*_abs_ in eV) was empirically corrected from the following equation (obtained for 13 microbial rhodopsins; coefficient of determination *R*^2^ = 0.920) (Tsujimura and Ishikita, 2021).

(Equation 4) $$E_{abs}=1.754\;E_{\text{TD}-\text{DFT}}-2.073$$

*ii) CASSCF/CASPT2.* The excitation energies and the energy levels of the MOs were calculated using the complete active space self-consistent field (CASSCF) with the second-order perturbation theory (CASPT2). 10 active electrons and 9 active orbitals were considered for photoactive yellow protein (i.e. CASSCF(10,9)/CASPT2), and 12 active electrons and 12 active orbitals were considered for KR2 (i.e. CASSCF(12,12)/CASPT2). The 6-31G∗∗ (photoactive yellow protein) or 6-31G∗ (KR2) basis sets was employed using the GAMESS program (Schmidt et al., 1993).

A QM/MM approach with the polarizable continuum model (PCM) method with a dielectric constant of 78 for the bulk region was employed to calculate the absorption energies. In this region, electrostatic and steric effects created by a protein environment were explicitly considered in the presence of bulk water. In the PCM method, the polarization points were placed on the spheres with a radius of 2.8 Å from the center of each atom to describe possible water molecules in the cavity. Radii of 2.8–3.0 Å from each atom’s center and dielectric constant values of ∼80 are expected to be optimal for the reproduction of excitation energetics, as evaluated in the polarizable QM/MM/PCM approach (Tamura et al., 2020; Tsujimura and Ishikita, 2021).

## Data Availability

The published article includes all datasets generated or analyzed during this study. This study did not generate new code. Any additional information required to reanalyze the data reported in this paper is available from the lead contact upon request.
