# Supplementary material for: Absorption wavelength along chromophore low-barrier hydrogen bonds
Source: iScience. 2022 Apr 13;25(5):104247. doi: 10.1016/j.isci.2022.104247 (PMC9062252; doi:10.1016/j.isci.2022.104247)
Supplement: Document S1. Figures S1–S8 and Tables S1 and S2 [file mmc1.pdf]

iScience, Volume *25*

Supplemental information

Absorption wavelength along chromophore low-barrier hydrogen bonds

Masaki Tsujimura, Hiroyuki Tamura, Keisuke Saito, and Hiroshi Ishikita

Supporting Information

Absorption wavelength along chromophore low-barrier hydrogen bonds

Masaki Tsujimura [1], Hiroyuki, Tamura [2,3], Keisuke Saito [2,3], Hiroshi Ishikita [2,3]*

1) Department of Advanced Interdisciplinary Studies, The University of Tokyo, 4-6-1 Komaba, Meguro-ku, Tokyo 153-8904, Japan

2) Department of Applied Chemistry, The University of Tokyo, 7-3-1 Hongo, Bunkyo-ku, Tokyo 113-8654, Japan

3) Research Center for Advanced Science and Technology, The University of Tokyo, 4-6-1 Komaba, Meguro-ku, Tokyo 153-8904, Japan

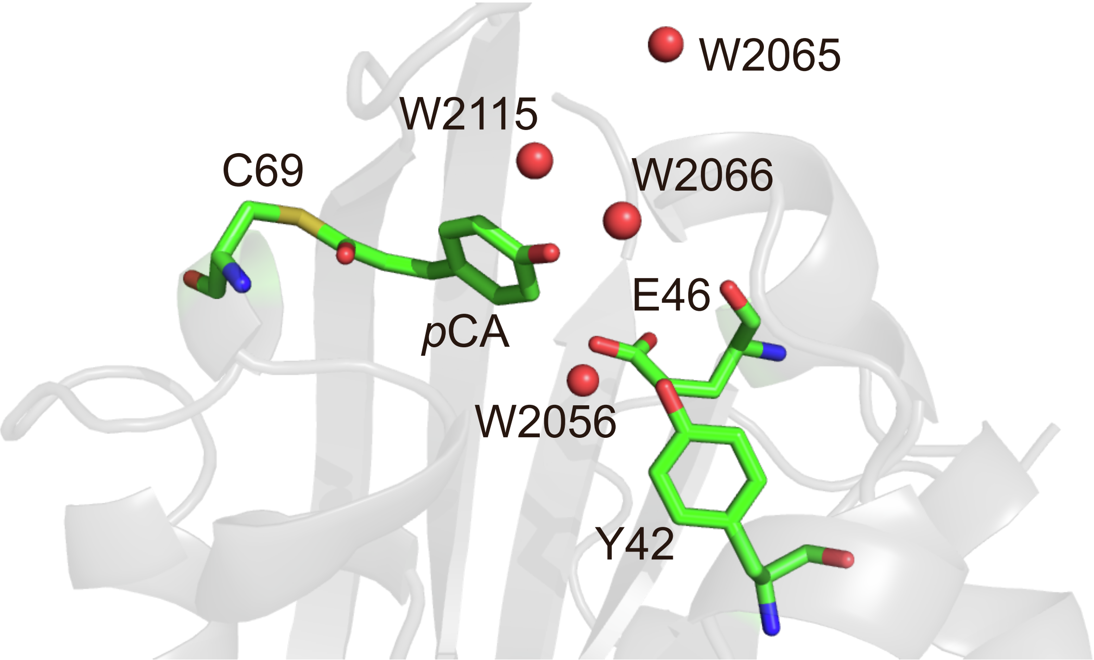

Figure S1. Crystal water molecules included explicitly for the calculation of pB_0 structure (PDB ID 4BBV (Schotte et al., 2012)), Related to **STAR Methods**. W2056 is included in the QM region, whereas W2065, W2066, and W2115 are included in the MM region.

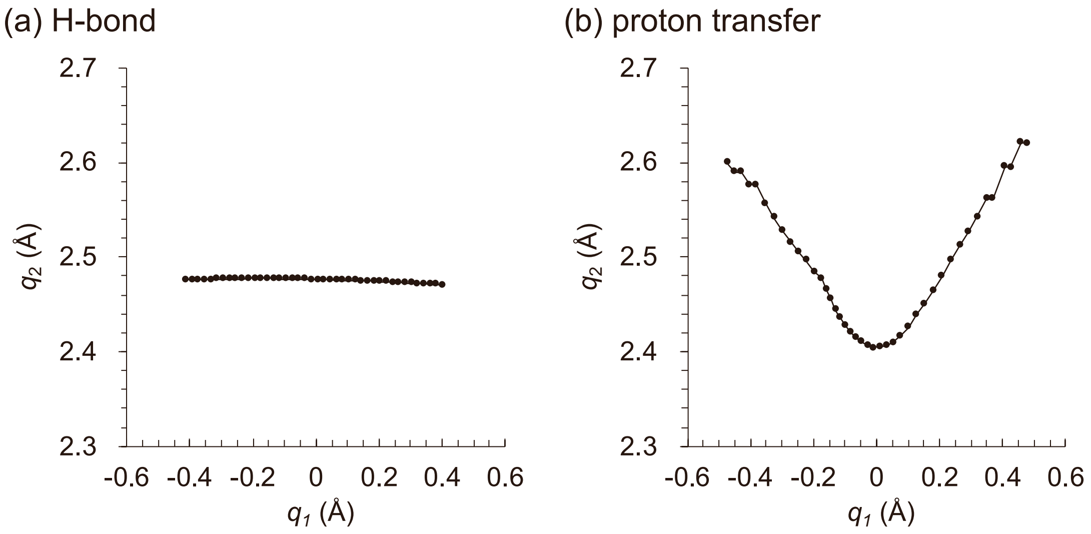

Figure S2. Correlation between q_1 and q_2 obtained for the low-barrier H-bond in the pR_{CW} state of photoactive yellow protein, Related to **Figure 4**. (a) H-bond (i.e., the H-bond donor…acceptor distance remains unchanged in response to the H^+ movement). (b) Proton transfer (i.e., the H-bond donor…acceptor distance changes in response to the H^+ movement). $q_1 = 0.5\ [r(O_{Glu46}...H) - r(H...O_{pCA})]$, $q_2 = r(O_{Glu46}...H) + r(H...O_{pCA})$, where r denotes the distance between the two atoms.

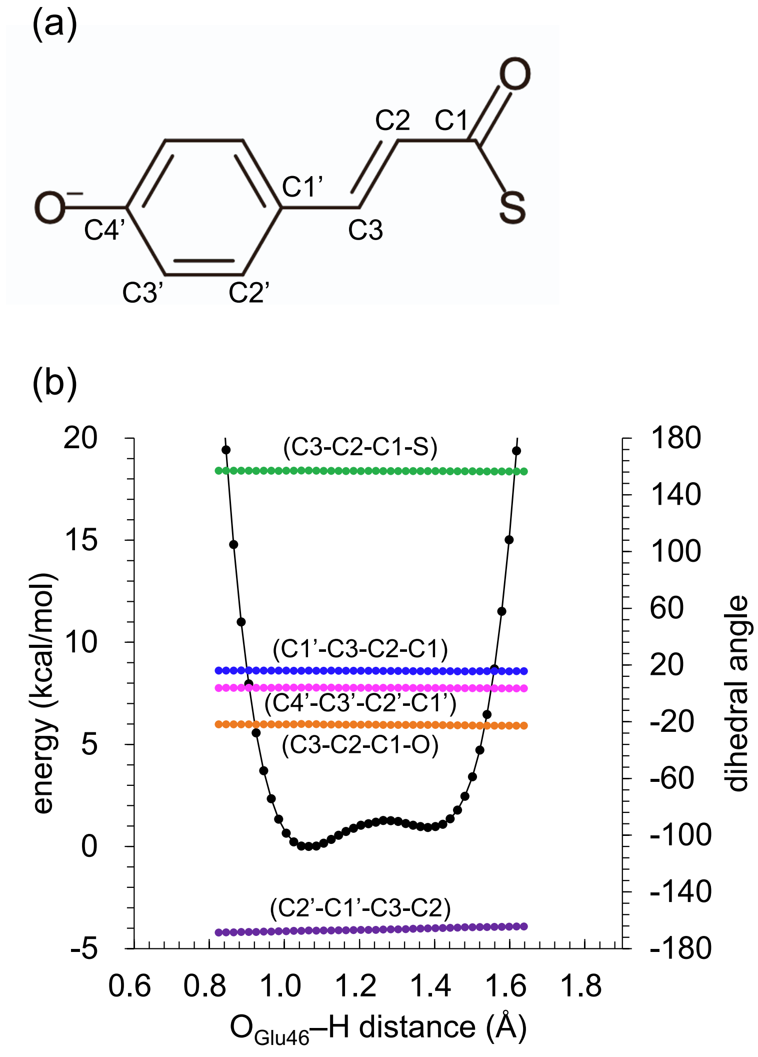

Figure S3. Changes in the dihedral angles (C3-C2-C1-S, C1’-C3-C2-C1, C4’-C3’-C2’-C1’, and C3-C2-C1-O) in the *p*CA region of the pR_{CW} structure, Related to **Figure 5b**.

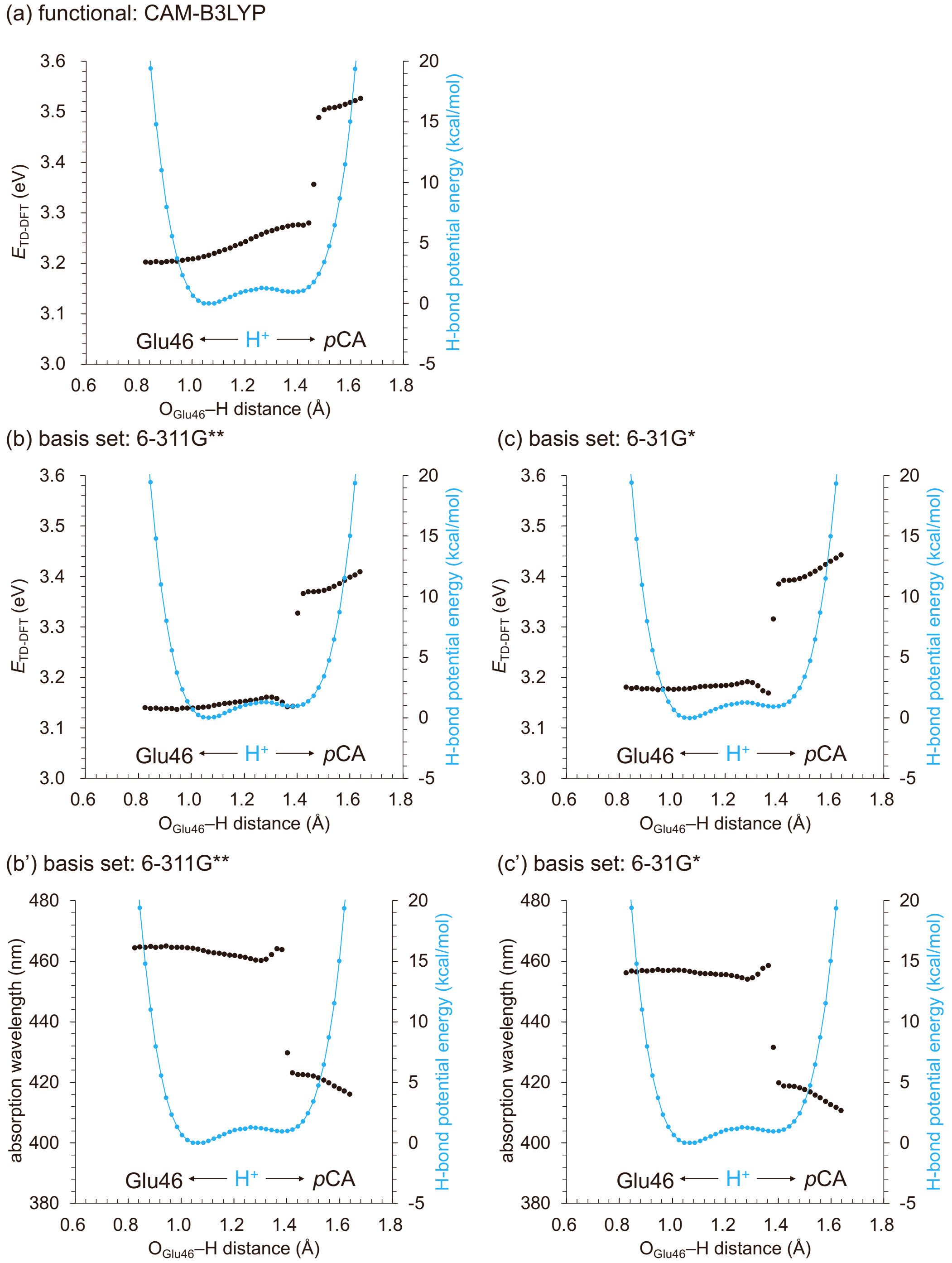

Figure S4. Absorption wavelength of *p*CA along the low-barrier H-bond in the pR_{CW} structure calculated using (a) the CAM-B3LYP functional (Yanai et al., 2004) (μ = 0.14), (b) 6-311G** (left vertical axis in eV), (c) 6-31G* (left vertical axis in eV), (b’) 6-311G** (left vertical axis in nm), and (c’) 6-31G* basis sets (left vertical axis in nm), Related to **Figure 5b**. Black circles indicate absorption wavelengths, and cyan balls indicate H-bond energies.

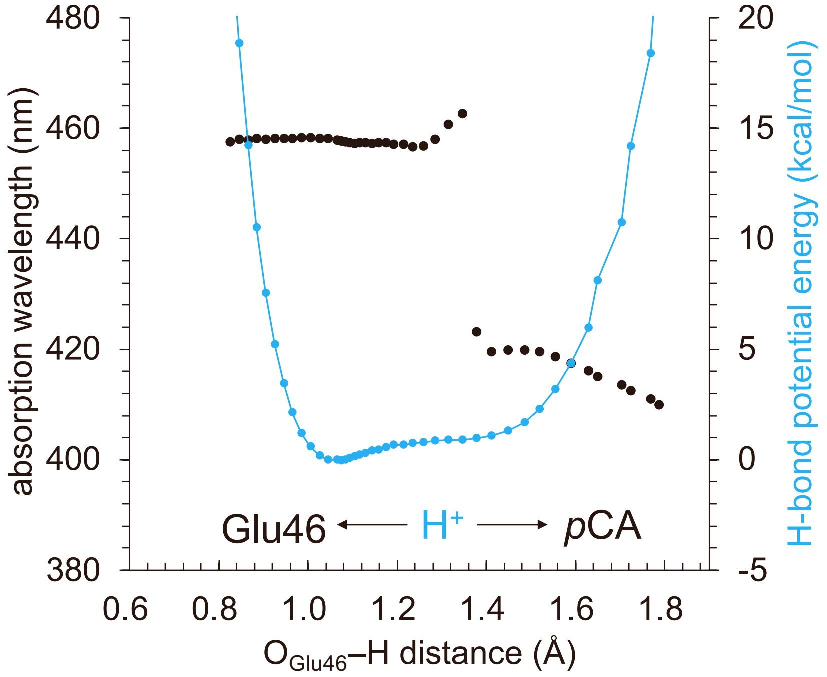

Figure S5. Potential energy profile for proton transfer (i.e., the H-bond donor and acceptor distance changes in response to the H^+ movement) and the absorption wavelength of *p*CA in the pR_{CW} state of photoactive yellow protein calculated using the TD-DFT method, Related to **Figure 5b**. Black circles indicate absorption wavelengths. Cyan balls indicate the energies.

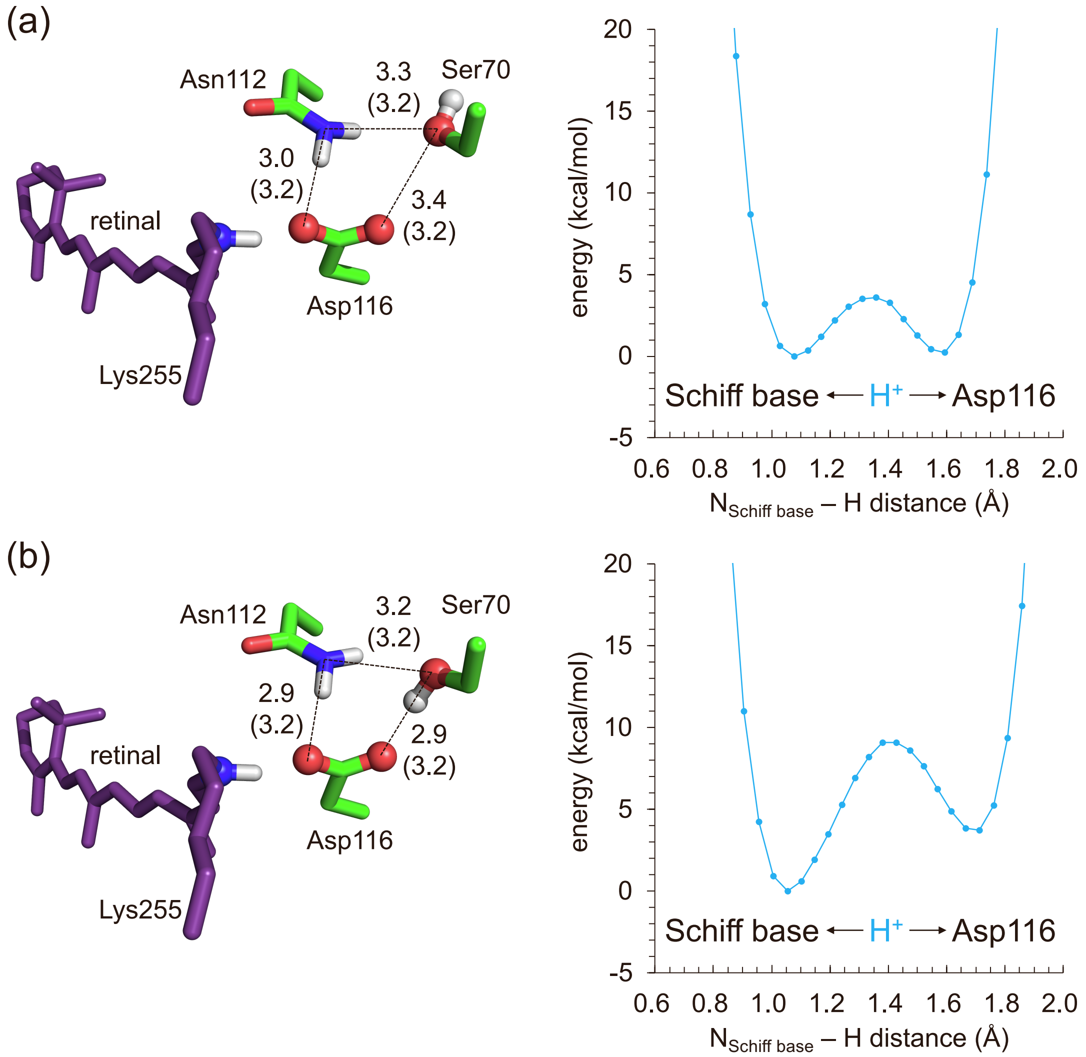

Figure S6. H-bond geometry of Asp116 (left panels) and energy profile (right panels) obtained for the time-resolved XFEL structure of KR2 (PDB ID 6TK3 (Skopintsev et al., 2020)), Related to **Figure 7**. (a) Ser70 does not donate an H-bond to Asp116. (b) Ser70 donates an H-bond to Asp116. Values in the left panels indicate distances in the QM/MM-optimized structure (in Å). Values in parentheses indicate distances in the crystal structure (Skopintsev et al., 2020).

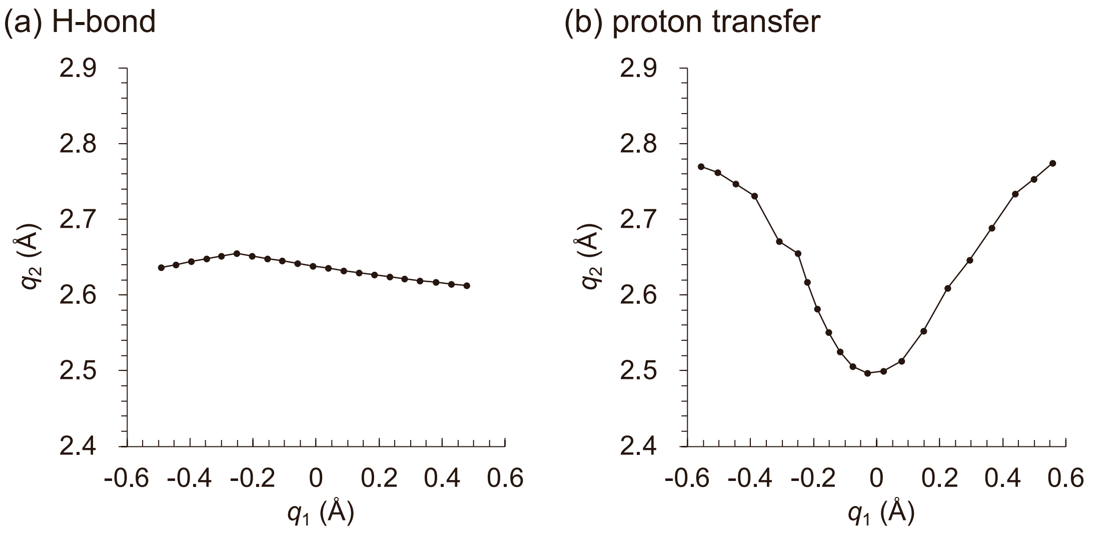

Figure S7. Correlation between q_1 and q_2 obtained for the time-resolved XFEL structure of KR2 (Skopintsev et al., 2020), Related to **Figure 7**. (a) H-bond (i.e., the H-bond donor…acceptor distance remains unchanged in response to the H^+ movement). (b) Proton transfer (i.e., the H-bond donor…acceptor distance changes in response to the H^+ movement). $q_1 = 0.5\ [r(N_{Schiff\ base}...H) - r(H...O_{Asp116})]$, $q_2 = r(N_{Schiff\ base}...H) + r(H...O_{Asp116})$, where r denotes the distance between the two atoms.

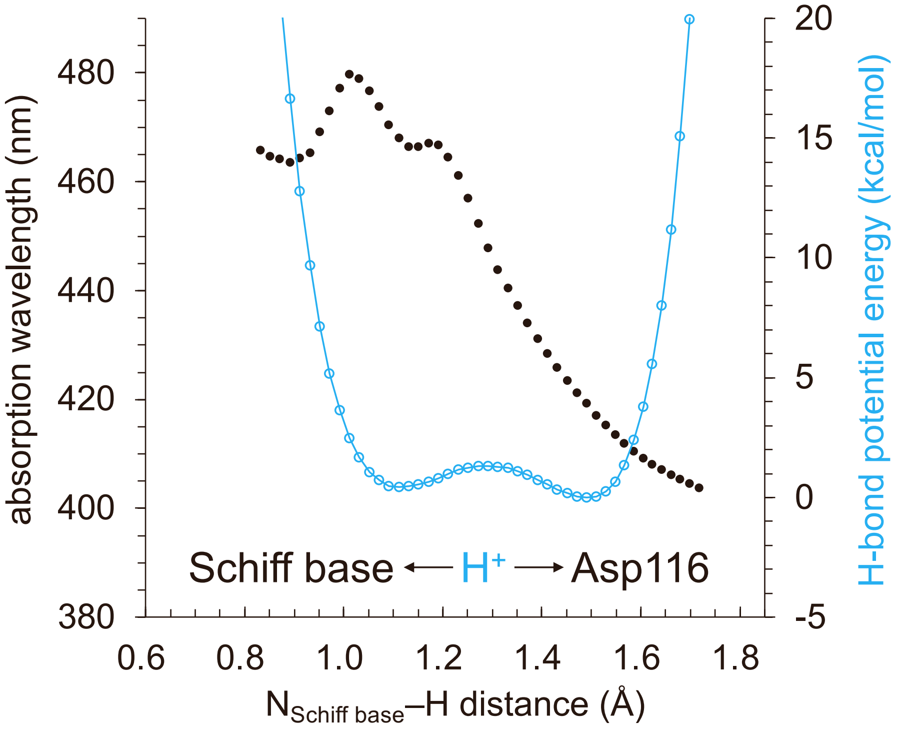

Figure S8. Absorption wavelength of the retinal Schiff base along the low-barrier H-bond in the low-pH structure (crystalized at pH 4 and soaked at pH 8) of KR2 (Kato et al., 2015) calculated using the TD-DFT method, Related to **Figure 8**. Black circles indicate absorption wavelengths and cyan open circles indicate H-bond energies (Tsujimura and Ishikita, 2021).

Table S1. The O_{Glu46}...H, H...O_{pCA}, and O_{Glu46}...O_{pCA} distances for H-bonds (i.e., the H-bond donor…acceptor distance remains unchanged in response to the H^+ movement) and proton transfer (i.e., the H-bond donor…acceptor distance changes in response to the H^+ movement) in the pR_{CW} state of photoactive yellow protein (in Å), Related to **Figure 4**.

**H-bond**			**proton transfer**		
**O_{Glu46}...H**	**H...O_{pCA}**	**O_{Glu46}...O_{pCA}**	**O_{Glu46}...H**	**H...O_{pCA}**	**O_{Glu46}...O_{pCA}**
1.62	0.85	2.46	1.77	0.85	2.60
1.58	0.89	2.46	1.70	0.89	2.57
1.54	0.93	2.46	1.63	0.93	2.55
1.50	0.97	2.46	1.55	0.97	2.51
1.46	1.01	2.46	1.48	1.01	2.49
1.42	1.05	2.47	1.41	1.05	2.46
1.38	1.09	2.47	1.35	1.09	2.43
1.34	1.13	2.47	1.28	1.13	2.41
1.30	1.17	2.47	1.23	1.17	2.40
1.26	1.21	2.47	1.19	1.21	2.40
1.22	1.25	2.47	1.16	1.25	2.41
1.18	1.29	2.47	1.13	1.29	2.42
1.14	1.33	2.47	1.10	1.33	2.43
1.10	1.37	2.47	1.08	1.37	2.45
1.06	1.41	2.47	1.06	1.41	2.47
1.02	1.45	2.47	1.02	1.47	2.49
0.98	1.49	2.47	0.98	1.53	2.51
0.94	1.53	2.47	0.94	1.60	2.54
0.90	1.57	2.47	0.90	1.67	2.57
0.86	1.61	2.47	0.86	1.73	2.58
0.82	1.65	2.47	0.82	1.77	2.59

Table S2. The $N_{Schiff\ base}$ (N_{SB})...H, H...O_{Asp116}, and N_{SB}...O_{Asp116} distances for H-bonds (i.e., the H-bond donor…acceptor distance remains unchanged in response to the H^+ movement) and proton transfer (i.e., the H-bond donor…acceptor distance changes in response to the H^+ movement) in the intermediate structure of KR2 (in Å), Related to **Figure 7**.

H-bond			proton transfer		
**N_{SB}...H**	**H...O_{Asp116}**	**N_{SB}...O_{Asp116}**	**N_{SB}...H**	**H...O_{Asp116}**	**N_{SB}...O_{Asp116}**
1.78	0.83	2.59	1.95	0.83	2.76
1.74	0.88	2.59	1.87	0.88	2.73
1.69	0.93	2.59	1.81	0.93	2.71
1.64	0.98	2.59	1.71	0.98	2.66
1.59	1.03	2.59	1.62	1.03	2.61
1.55	1.08	2.59	1.53	1.08	2.58
1.50	1.13	2.59	1.42	1.13	2.52
1.45	1.18	2.59	1.33	1.18	2.48
1.40	1.23	2.59	1.27	1.23	2.46
1.36	1.28	2.59	1.22	1.28	2.46
1.31	1.33	2.60	1.18	1.33	2.47
1.26	1.38	2.60	1.15	1.38	2.49
1.22	1.43	2.60	1.12	1.43	2.51
1.17	1.48	2.60	1.10	1.48	2.54
1.12	1.53	2.61	1.09	1.53	2.57
1.08	1.58	2.61	1.08	1.58	2.61
1.03	1.62	2.60	1.03	1.64	2.62
0.98	1.67	2.60	0.98	1.75	2.67
0.93	1.72	2.60	0.93	1.82	2.69
0.88	1.76	2.60	0.88	1.88	2.70
0.83	1.81	2.60	0.83	1.94	2.71
